# Microbes-mediated integrated nutrient management for improved rhizo-modulation, pigeonpea productivity, and soil bio-fertility in a semi-arid agro-ecology

**DOI:** 10.3389/fmicb.2022.924407

**Published:** 2022-09-15

**Authors:** Gaurendra Gupta, Shiva Dhar, Adarsh Kumar, Anil K. Choudhary, Anchal Dass, V. K. Sharma, Livleen Shukla, P. K. Upadhyay, Anup Das, Dinesh Jinger, Sudhir Kumar Rajpoot, Manjanagouda S. Sannagoudar, Amit Kumar, Ingudam Bhupenchandra, Vishal Tyagi, Ekta Joshi, Kamlesh Kumar, Padmanabh Dwivedi, Mahendra Vikram Singh Rajawat

**Affiliations:** ^1^Indian Grassland and Fodder Research Institute, Indian Council of Agricultural Research, Jhansi, India; ^2^Division of Agronomy, Indian Agricultural Research Institute, Indian Council of Agricultural Research, New Delhi, India; ^3^National Bureau of Agriculturally Important Microorganisms, Indian Council of Agricultural Research, Mau, India; ^4^Central Potato Research Institute, Indian Council of Agricultural Research, Shimla, India; ^5^Division of SSAC, Indian Agricultural Research Institute, Indian Council of Agricultural Research, New Delhi, India; ^6^Division of Microbiology, Indian Agricultural Research Institute, Indian Council of Agricultural Research, New Delhi, India; ^7^Research Complex for NEH Region, Tripura Centre, Indian Council of Agricultural Research, Lembucherra, India; ^8^Indian Institute of Soil and Water Conservation, Research Centre, Indian Council of Agricultural Research, Vasad, India; ^9^Institute of Agricultural Sciences, Banaras Hindu University, Varanasi, India; ^10^ICAR RC for NEH Region, Sikkim Centre, Gangtok, India; ^11^ICAR–Krishi Vigyan Kendra, ICAR RC for NEH Region, Manipur Centre, Tamenglong, India; ^12^Indian Institute of Seed Science, Indian Council of Agricultural Research, Mau, India; ^13^Rajmata Vijayaraje Scindia Krishi Vishwa Vidyalaya, Gwalior, India; ^14^Indian Institute of Farming Systems Research, Indian Council of Agricultural Research, Meerut, India; ^15^Department of Plant Physiology, Institute of Agricultural Sciences, Banaras Hindu University, Varanasi, India

**Keywords:** crop growth, microbial inoculants, nutrient management, pigeonpea, soil health, root growth

## Abstract

Excessive dependence on chemical fertilizers and ignorance to organic and microbial inputs under intensive cropping systems are the basic components of contemporary agriculture, which evolves several sustainability issues, such as degraded soil health and sub-optimal crop productivity. This scenario urges for integrated nutrient management approaches, such as microbes-mediated integrated plant nutrition for curtailing the high doses as chemical fertilizers. Rationally, experiment has been conducted in pigeonpea at ICAR-IARI, New Delhi, with the aim of identifying the appropriate nutrient management technique involving microbial and organic nutrient sources for improved rhizo-modulation, crop productivity, and soil bio-fertility. The randomized block-designed experiment consisted nine treatments *viz*. Control, Recommended dose of fertilizers (RDF), RDF+ Microbial inoculants (MI), Vermicompost (VC), Farm Yard Manure (FYM), Leaf Compost (LC), VC + MI, FYM + MI, and LC + MI. *Rhizobium* spp., *Pseudomonas* spp., *Bacillus* spp., and *Frateuria aurantia* were used as seed-inoculating microbes. The results indicated the significant response of integration following the trend VC + MI > FYM + MI > LC + MI > RDF + MI for various plant shoot-root growth attributes and soil microbial and enzymatic properties. FYM + MI significantly improved the water-stable aggregates (22%), mean weight diameter (1.13 mm), and geometric mean diameter (0.93 mm), soil organic carbon (SOC), SOC stock, and SOC sequestration. The chemical properties *viz*. available N, P, and K were significantly improved with VC + MI. The study summarizes that FYM + MI could result in better soil physico-chemical and biological properties and shoot-root development; however; VC + MI could improve available nutrients in the soil and may enhance the growth of pigeonpea more effectively. The outcomes of the study are postulated as a viable and alternative solution for excessive chemical fertilizer-based nutrient management and would also promote the microbial consortia and organic manures-based agro-industries. This would add to the goal of sustainable agricultural development by producing quality crop produce, maintaining agro-biodiversity and making the soils fertile and healthy that would be a “gift to the society.”

## Introduction

With the ever-growing population, scarcity of arable lands, and resources alongside multiple environmental pressure, the need to focus on efficient natural resources management in agriculture for food security and sustainability is one of the biggest challenges for researchers and farming community (Meena et al., 2021). To meet this challenge, environment-unfriendly agricultural chemicals, such as fertilizers, have played a significant role in green revolution and are even, today, commonly recommended to outwit nutrient deficiencies of the soils (Kumar et al., 2018a). The use of agro chemicals has been a major factor in improving crop production; however, it causes severe deterioration in soil health and quality and thus poses a negative impact on crop production sustainability and environmental safety (Nelson et al., 2019). Besides this, fertility exhaustive cereal-based cropping systems are also having serious implications on sustainability of the agro-eco system (Gupta et al., 2018, 2020; Tanveer et al., 2019). The disruption of microbial diversity and, consequently, the bio-fertility, owing to the inappropriate nutrient management strategy, has become a concern for optimum soil health and plant growth (Fernández-Romero et al., 2016; Patra et al., 2016). In this context, the role of naturally abundant but functionally unexplored microorganisms, such as nitrogen fixing, phosphorus, and potash solubilizing microbial inoculants, along with their synergic integration with other organic sources of nutrients, such as bulky manures under a fertility augmentative legume crop, is crucial and offers a unique opportunity for balancing soil fertility, maintaining soil health, improving plant growth and profitable crop production in a sustainable way (Kumar et al., 2015; Gupta et al., 2020; Bhakar et al., 2021).

The determination of soil health and quality parameters, along with assessing the influence on the plant root-shoot growth attributes, is an important aspect of assessing a soil-plant management technique; and their precise estimates are essential to judge the impact of any soil fertility management experiment on the agricultural productivity and sustainability (Allen et al., 2011; Thapa et al., 2017; Bünemann et al., 2018; Nath et al., 2018; Miner et al., 2020).

Imbalanced fertilizer use and mismanagement of crop residue *via* burning and removal from the field are still in practice under cereal-cereal cropping systems (Kannan et al., 2013; Kumar et al., 2018a; Kumar S. et al., 2021), which cause soil and environmental pollution, nutrient mining, multi-nutrient deficiency, losses in soil bio-fertility, the reduction in partial factor productivity, loss of agri-biodiversity, and, ultimately, poor soil health and sub-optimal crop production (Bastida et al., 2006; Mahetele and Kushwaha, 2011). Considering these negative implications on agro-ecology, the need for reduced and balanced fertilizer applications, along with crop diversification, is felt more essential than ever before (Thapa et al., 2017; Kumar et al., 2018a). “Legumes in crop rotation” are considered as an effective and much-needed approach to diversifying problematic cropping systems, (CS) such as rice-wheat CS (Ghosh et al., 2020). In this regard, pigeonpea, by virtue of its superior physiological and agronomical traits, makes it hardy and adaptive, has emerged as the best alternate in the *kharif* season in India to replace the rice crop, which is nutrient and water exhaustive for semiarid agro-ecosystem (Fernández-Romero et al., 2016). Besides, integrated nutrient management (INM) for balanced nutrition is also proved worthy (Jinger et al., 2020, 2021) and much-needed strategy under present circumstances and scenarios of semiarid regions of India (Song et al., 2015; Singh et al., 2019, 2020b). The practice of high, rather balanced, input application does not need fulensue in the growth of the agriculture sector with sustainability point of view, especially in respect of optimized crop production and resource use (Mahetele and Kushwaha, 2011); whereas, soil with good health and quality driven by balanced input management would be helpful to produce higher crop production in a sustainable manner under varied agro-climatic conditions (Congreves et al., 2015). In this view, microbial inoculants and organic manure-based INM technique could minimize the undesirable repercussions, resulting from cultivation of exhaustive crops, intensive cultivation, and imbalanced use of fertilizers followed through the restriction on use of organic manures and microbial inoculants (Samal et al., 2017).

Nowadays, microbial inoculants cultures are considered as a key component of plant nutrient management and fertility restoration that enhance soil microbial health, maintain soil fertility, improve crop growth and productivity thus make a contribution to sustainable agro-ecosystems (Harish et al., 2022; Kumar et al., 2022). It is a component that aggregates an expansion of microbes-based bio-products primarily, whose bioactivities are vital to stimulate and enhance the biological functions of intricate soil-microbe-plant continuum (Singh et al., 2019). The combined use of mineral, organic, and microbial resources is an emerging researchable area that targets to develop and design coherent microbes-based techniques, which are highly amicable with inorganic and organic inputs, with positive impacts on soil, crops, and environment (Gupta et al., 2020). Integration of microbial inoculants, along with chemical fertilizers and organic resources of nutrients, could improve soil health, plant growth, and thus sustain the soil and crop productivity (Singh et al., 2018, 2020a,^2021^). Microbial inoculants-based nutrient management is a sustainable and eco-friendly strategy since it uses available forms of organic and inorganic nutrients to develop healthy environmental conditions and economically viable crop production systems, besides their plant growth-promoting (PGP) ability (Dhandayuthapani et al., 2015; Gupta et al., 2020). Despite its utmost importance, very meager research reports are available that advocate direct evidence of linking greater soil organic matter (SOM) resulting from microbial inoculants and manure-based INM to increase the soil health, root development and growth of crops, and much work remains to be done to find out the impact of such INM practices with respect to soil-plant relationship (Gupta et al., 2020, 2018; Lal, 2020). The exploration of microbial inoculants and organic manure-based technique is also believed to have the impact on the agro-industry by promoting the microbial inoculants culture-based agri-startups and organic manure-based agri-business. Outcomes of the study are also expected to solve the problem of crop residue and animal bi-products management in form of its use as manure for soil health and fertility augmentation. Overall, the study has the linkage with the various concurrent intangible ecological and social aspects apart from measurable impact on agro-biodiversity and production sustainability. Considering the rational, the study with an objective of assessing the influence of microbes mediated integrated nutrient management on root-shoot growth-promoting ability, soil health, rhizosphere modulation, and growth parameters of pigeonpea was conducted to bring an appropriate and biologically viable and integrated recommendation for efficient nutrient management for enhanced crop production besides maintaining soil fertility and health.

## Materials and methods

### Experiment allocation, agro-climate, and soil characteristics

**FIGURE 1:**
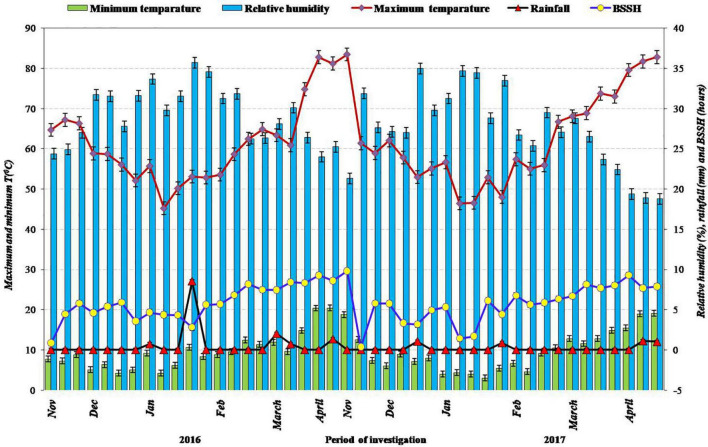
Meteorological data during the period of investigation during 2016 and 2017, respectively.

The 2-year field experimentation on a fixed site was done at ICAR-Indian Agricultural Research Institute, New Delhi (28.38°N Latitude, 77.09°E Longitude, 229 m Altitude) during *kharif* 2016 and *kharif* 2017. The climate of the experimental site is semiarid type and subtropical in nature; hot winds prevail during the summer season and severe cold during the winter season. Mean maximum temperature during the period of crop cultivation was 34.3 and 33.5°C; mean minimum temperature, 22.8 and 22.1°C; total rainfall was 665.8 and 707.4 mm during 2016 and 2017, respectively. Average annual pan evaporation was recorded as about 850 mm. The details of meteorological observations throughout the field experimentation period (*Kharif*, 2016 and *Kharif*, 2017) are depicted in Figure 1. The soil of the research experiment site was alluvial, sandy clay loam in texture, low in SOC content (0.40%) and available nitrogen (164.5 kg ha^–1^), medium in available phosphorus (14.5 kg ha^–1^), high in available potassium (292.5 kg ha^–1^), and slightly alkaline in reaction. The physical, chemical, and biological characteristics of soil analyzed from the initial soil test are described in Table 1.

**TABLE 1:** Physico-chemical and biological properties of the experimental field (initial value).

SN	Particulars	Values		References
		
		2016	2017	
I	Mechanical analysis			
	Sand (%)	63.28	63.40	Bouyoucos, 1962
	Silt (%)	12.24	12.26	
	Clay (%)	24.48	24.34	
	Textural class	Sandy clay loam		
II	Physical Properties			
	Bulk density (Mg m^–3^)	1.58	1.56	Piper, 1966
	Water stable aggregates (%)	11.5	11.0	Yoder, 1936; Bouyoucos, 1962
III	Chemical properties			
	Soil organic carbon (%)	0.39	0.40	Jackson, 1973
	Available N (kg ha^–1^)	164.5	166.5	Subbiah and Asija, 1956
	Available P (kg ha^–1^)	14.5	14.8	Olsen et al., 1954
	Available K (kg ha^–1^)	292.0	292.0	Jackson, 1973
	pH (1:2.5, soil: water)	7.8	7.8	Jackson, 1973
	Electrical conductivity (EC, dsm^–1^)	0.40	0.39	Jackson, 1973
IV	Biological properties			
	Microbial biomass carbon (MBC, mg kg^–1^ soil)	102.5	112.0	Nunan et al., 1998
	Dehydrogenase activity (DHA, μg TPF g^–1^ soil day^–1^)	105.5	118.0	Casida et al., 1964
	Alkaline phosphatase activity (APA, μg PNP g^–1^ soil hr^–1^)	46.2	50.3	Tabatabai and Bremner, 1969
	Acetylene reduction activity (ARA, n moles ethylene gram^–1^ soil hr^–1^)	13.5	16.8	Rakshit et al., 2018
	Total polysaccharides (mg kg^–1^ soil)	266.5	270.8	Acton et al., 1963

### Experimental treatments and design

The present study was designed in randomized block design (RBD) replicated three times and consisted of nine treatments of nutrient management, *viz*. Control, recommended dosage of fertilizers (RDF, 30: 60: 40 kg NPK ha^–1^), RDF+ microbial inoculants (MI), Vermicompost (VC), Farm Yard Manure (FYM), Leaf Compost (LC), VC + MI, FYM + MI, and LC + MI. The details of treatments under experimentation are depicted in Table 2.

**TABLE 2:** Treatment details of the experiment.

T_1_	Control	T_6_	LC (5 t ha^–1^)
T_2_	RDF (30:60:40 kg N:P:K ha^–1^)	T_7_	VC (5 t ha^–1^) + MI
T_3_	RDF + Microbial inoculants (MI)	T_8_	FYM (5 t ha^–1^) + MI
T_4_	VC (5 t ha^–1^)	T_9_	LC (5 t ha^–1^) + MI
T_5_	FYM (5 t ha^–1^)		

### Microbial inoculants and organic manures

The *Rhizobium leguminosarum* as nitrogen-fixing inoculants, phosphorus-solubilizing bacteria (PSB) (*Pseudomonas striata, Pseudomonas putida, Bacillus megaterium*, and *Bacillus subtilis*) as phosphorus-solubilizing microbial inoculants, and *Frateuria aurantia* and *Bacillus mucilagenosus* as potassium solubilizers were used for seed inoculation. Seed treatment with *Rhizobium* and PSB was done at the rate of 250-g carrier-based culture 10^–1^ kg seed and K-solubilizing microbes (liquid-based formulation) at the rate of 50-ml culture 10^–1^ kg seeds. The slurry method of seed treatment was used for proper mixing of microbial inoculants culture and seeds. After the mixing, the seed was allowed to dry in a cool and shady area before sowing. Organic manures used under the experiment were vermicompost, FYM, and leaf compost. These manures were analyzed for their nutrient concentration, which is presented in Table 3.

**TABLE 3:** Chemical composition of FYM, vermicompost, and leaf compost used in the experiment.

Nutrient content	FYM		Vermicompost		Leaf compost	
			
	2016	2017	2016	2017	2016	2017
Total N (%)	0.52	0.57	1.48	1.45	0.40	0.40
Available P (%)	0.20	0.25	0.51	0.47	0.15	0.16
Available K (%)	0.55	0.56	1.37	1.40	0.45	0.44
Fe (mg kg^–1^)	1700	1730	2024	1991	1400	1350
Zn (mg kg^–1^)	107.5	104.2	100.2	99.6	85.5	95.5
Mn (mg kg^–1^)	341.7	354.2	370.1	354.8	320.5	332.5
Cu (mg kg^–1^)	15.8	16.2	17.7	18.1	15.0	15.5

### Crop management

The seeds of pigeonpea variety Pusa-991 (an early maturing short duration variety, which fits well under pigeonpea-wheat cropping system of IGP) were seeded at 60 cm (row-row) × 20 cm (plant-plant). Seed sowing was done manually at the depth of 5 cm by using a 15-kg ha^–1^ seed rate. At the stage of field preparation, the fertilizer inputs (30:60:40 kg N, P_2_O_5_, and K_2_O ha^–1^), FYM (5 t ha^–1^), VC (5 t ha^–1^), and LC (5 t ha^–1^) were applied as per treatment protocol. NPK fertilizers were applied as the basal through urea, diammonium phosphate (DAP) and muriate of potash (MoP), respectively. A total of three irrigations were scheduled at the branching, flowering, and pod-filling stages. The preliminary use of pendimethalin 30 EC @.9 kg ha^–1^, along with one manual weeding at 30 DAS, was practiced for the management of weed in crops. Dimethoate 30 EC (Rogor) at the rate of 250-ml a.i. ha^–1^ (0.025%) and monocrotophos at the rate of 300-ml a.i. ha^–1^ (0.03%) were introduced as spray to control insect-pests of the crop. Zineb (0.2%) was applied at 60 DAS to control fungal diseases like the leaf spot. The crop was harvested when seeds became relatively hard, having 75–80% moisture. The harvested produce from the net plot area was sundried for 4–5 days; after that, bundle weight was recorded. Pullman thresher was used for threshing and seed cleaning.

### Data recording and analysis

#### Crop growth parameters

Growth parameters were observed and recorded as per standard methodology and protocols. Plant height was taken from the base region to the tip of the longest branch at 30, 60, and 90 days after sowing (DAS) and at the maturity stage. The plant dry matter accumulation (DMA) was noted at a regular interval of 30, 60, and 90 DAS and at the maturity stage. For this purpose, the harvesting of plants of a 0.5-m continuous row length area was done at different growth stages. The observations on number of the branches plant^–1^ were recorded by counting the five tagged plants of each plot at maturity. Then, the average was calculated and value expressed as a number of the branches plant^–1^. After measuring the leaf area, these plants were kept for sun-drying and then oven-drying at 65°C till the constant weight was achieved. Finally, DMA value was expressed as g plant^–1^. The leaf area of randomly selected five plants from each experimental plot was noted at the 30-, 60-, and 90-DAS stage using the leaf area meter. Then, the average was calculated, and the final value of the leaf area was expressed in the cm^2^ plant^–1^. Leaf area index (LAI) was worked out by dividing value of the leaf area with the ground area using the following standard formula:


Leafareaindex(LAI)=Leafarea(sq.cm)/Landarea(sq.cm)

The mean crop growth rate of the crop was estimated using the following formula:


$$\text{CGR}=\left(\frac{{\text{W}}_{2}-{\text{W}}_{1}}{{\text{T}}_{2}-{\text{T}}_{1}}\right)\,\left(\frac{1}{\text{S}}\right)$$

where

W_1_ and W_2_ represent dry weight (g) at time T_1_ and T_2_, respectively

T_2_- T_1_ represent the interval of time (days)

S is the value of the land area (m^–2^) as occupied by crop plants

CGR of the crop expressed as g m^–2^ of the land area day^–1^.

#### Soil health parameters

Soil physical parameters *viz.*, Bulk density (BD), water stable aggregates (WSA), mean weight diameter (MWD), and geometric mean diameter (GMD) were recorded at the beginning and the end of the crop cycle. The sample for measuring field BD was collected by the core sampler of Piper (1966) from the randomly selected 3–4 areas of each experimental plot. The distribution of aggregate size was measured using approximately 250-g oven dried soil, and the remaining portion of soil was air-dried at the room temperature. Then, the material was passed through a 2-mm sieve in order to determine aggregate particle size distribution, following the Bouyoucos Hydrometer Method (Bouyoucos, 1962). The wet-sieving method was used for the estimation of the size distribution of WSA (Yoder, 1936). The GMD and MWD of soil aggregates were measured, with the method outlined by Kemper and Rosenau (1986). For separating the 4- to 8-mm aggregates from the bulk soil, the dry-sieving technique was used. During this process, the rupture of aggregates is common; hence, to prevent a sudden rupture, 50 g of aggregates was placed on the first sieve and moistened gently from below. Then, the set of sieves was shaken in water for about 10 min at the oscillations rate of 30 min^–1^. Now, after correction for sand content, the settled soil on each sieve was utilized for calculating the MWD of the WSA. The percentage weight of aggregates retained on sieves (>0.25-mm diameter) was noted as WSA. The calculation of WSA was worked out as per the following equation:


$$M\,W\,D=\sum_{i=1}^{n}\bar{X\,i}\,W\,i$$

$$G\,M\,D=\exp\left[\sum_{i=1}^{n}\log\bar{x\,i}\,w\,i/m\right]$$

where, ‘Xi’ represents the mean fraction diameter, ‘i’ is the aggregate diameter fraction, ‘Wi’ is the proportion of total sample weight in that particular fraction, and ‘m’ is the total sample mass.


$$\mathrm{Distribution}\mathrm{of}\mathrm{water}\mathrm{stable}\mathrm{aggregates}\left(\%\right)=\frac{\left(\mathrm{Aggregates}\,\mathrm{weight}\,\mathrm{in}\,\mathrm{each}\,\mathrm{size}\,\mathrm{group}\right)}{\left(\mathrm{Total}\,\mathrm{weight}\,\mathrm{of}\,\mathrm{soil}\right)}\times 100$$

Soil chemical characteristics (initial and end of the experiment) were estimated from collected soil samples from the depth of 0–15 cm of the soil profile. Soil reaction (pH) was analyzed in aqueous extract of soil and de-ionized water using the soil:water ratio as 1:2.5. The collected soil samples were air-dried and organic carbon (OC was measured by following the procedure described by Walkley and Black (Jackson, 1973). Available nutrients (N, P, and K) were estimated by soil sample collection from the depth of 0–15 cm of the soil profile from the experimental plot at the initial stage and the end of each experiment. The samples for N, P, and K analyses were air-dried, grounded, and allowed to pass through a 2-mm mesh sieve. The available N was analyzed through the alkaline potassium permanganate (KMnO_4_) procedure as described by Subbiah and Asija (1956) and the value expressed as kg ha^–1^. The available P status in soil was measured by following the Olsen’s method (Olsen et al., 1954) and expressed as kg ha^–1^. Available K was measured from the neutral ammonium acetate extraction method of Jackson (1973) and expressed as kg ha^–1^. On the basis of primary data (SOC and BD values), the SOC stock, the build-up rate, and the sequestration rate were calculated (Lenka et al., 2013; Paul et al., 2016; Yadav et al., 2018). The following formula has been used for calculating SOC stock and the rate of sequestration:


$$\mathrm{SOC}\,\mathrm{stock}\,\left(\text{Mg}/\text{ha}\right)=\frac{\begin{array}{c} \text{SOC}\times \mathrm{Bulk}\,\mathrm{density}\,\left(\mathrm{Mega}\,g/\mathrm{cubic}\,m\right)\\ \times \mathrm{Soil}\,\mathrm{depth}\,\left(\text{m}\right)\times 10000 \end{array}}{100}$$

The rate of soil carbon sequestration (Mg C ha^–1^ year^–1^) for the 2 years of the experimental period was estimated as a change in the soil organic carbon (SOC) stock (Mg ha^–1^) divided by the experiment duration (year).


$$\mathrm{Soil}\,C\,\mathrm{sequestration}\,\mathrm{rate}=\frac{\text{SOC}\,\left(\text{final}\right)-\text{SOC}\,\left(\text{initial}\right)}{\mathrm{Duration}\,\mathrm{of}\,\mathrm{experiment}}$$

The biological properties of soil were estimated by collecting the samples from the depth of 0–15 cm of the soil profile using the tube augur at the crop stage of 50% flowering (75 days after sowing). The gentle sieving by the use of a 4-mm mesh sieve was done to remove plant roots, stones, and large organic contents. After sieving, the field moist samples were subjected to pass by the use of a 2-mm sieve and laid in at 4°C until used for the estimation of soil microbial biomass carbon and activities of enzymes (dehydrogenase and alkaline phosphatase). Soil microbial biomass carbon (SMBC), dehydrogenase activity (DHA), and alkaline phosphatase activity (APA) in soil samples were measured by following the method described by Casida et al. (1964), Tabatabai and Bremner (1969), and Nunan et al. (1998) respectively. The brief methodology for the estimation of soil microbial parameters is as follows:

For the determination of SMBC (mg kg^–1^ soil), a 5-g soil sample was fumigated with chloroform and then incubation for 24 h. After the incubation process, the evaporation of chloroform was done at 50*^o^*C in Bio-chemical oxygen demand (BOD) by opening the tube caps for 20–24 h. After the process of evaporation, 70 ml of 0.5-M potassium sulfate (K_2_SO_4_) was added in samples and shaken for 30 min, followed by settle down. Final supernatant liquid was subjected for the measurement of absorbance. The final value of SMBC was worked out from the following formula:


SMBC⁢(mg⁢per⁢kg⁢soil)=Fumigated⁢soil⁢organic⁢carbon-Unfumigated⁢soil⁢organic⁢carbon×17.5×15487

For the estimation of DHA (μg TPF g^–1^ soil day^–1^), saturation of 6.0 g of the soil sample was done with 1.0-ml Tri phenyl tetrazolium chloride (TTC) by adding 0.1-g calcium carbonate (CaCO_3_) and incubated for a day; then, methanol (10 ml) was added, and the sample tube was shaken for about 1.0 min followed by filtration to obtain supernatant liquid. The absorbance reading of the supernatant liquid was taken by the Spectrophotometer at the wavelength of 485 nm. The final calculation was worked out with the following equation:


DHA(μgTPF/gsoil)/day)=Soil⁢weight⁢(g)×Concentration×Dilution⁢factor

Alkaline phosphatase activity (μg p-nitro phenol g^–1^ soil hr^–1^) was determined by estimating *p*-nitrophenol (PNP) as relinquished by incubation of a 1-g soil sample with 0.25-ml toluene, a 4-ml universal buffer, and a 1 ml of a 5-mM substrate at 37°C for 1 h. The content of *p*-nitro phenol in the sample was estimated from the Spectrophotometer at the wavelength of 440 nm.

For the measurement acetylene reduction activity in soil, estimation of ethylene (index of nitrogenase activity) with gas chromatography was done (Rakshit et al., 2018). The ARA values are expressed as moles of ethylene produced g^–1^ soil hr^–1^. For this purpose, a 2-m long Porapak-R stainless steel column and a flame ionization detector were used for the estimation of gas samples on Gas Chromatograph (GC) of Hewlett Packard 5890 series II. The column temperature was maintained at 100°C, and the injector and detector temperature were maintained at 110°C. A gas flow rate of 35-ml min^–1^ of N_2_ was served as carrier gas.


Soil⁢Acetylene⁢Reduction⁢Activity⁢(ARA)=0.1653×x×(conc.byG.C.)

The total polysaccharides content of soils as affected through different treatments was analyzed following the method as described by Acton et al. (1963). For the colorimetric measurement of the carbohydrate content in soil, 10 g of soil was disseminated in 10 ml of.5-N NaOH for 10 min in a Waring blender, moved in Erlenmeyer flasks by addition of 60-milliliter NaOH, and shaken for 3 h. The soil suspension was then maintained with the concentrated hydrochloric acid (HCI) up to a pH value of 2–3, and the fulvic acids were isolated from the humic acids and soil colloids by centrifugation process. The proportion of humic acids and soil colloids remaining in the centrifuged tube was then washed with the acidified water made by adding 10-ml concentrated HCI in 400-ml H_2_O. The process of centrifugation was done again, and the supernatant liquid was summed to the previous extract. The fulvic acids supernatant was then diluted to volume where two volumes of acetone were mixed to one volume of an aliquot of solution. The supernatant solution, after centrifuging, comprised the acetone soluble fraction. The removal of acetone was done by evaporation up to dryness using a steam bath, and the residues dissolved in water. The precipitated flocculent known as microbial gum was redissolved in 0.5 A/NaOH, and also volume was maintained. The fractions of organic matter were hydrolyzed through sufficient H_2_SO_4_ to provide a solution equivalent to 100 ml of 3 N H_2_SO_4_. Polysaccharide constituents were then estimated with anthrone.

### Root properties

For the measurement of root properties, samples of plant roots were collected from the third row of the cultivated crop at the flowering stage of the 50% (75 days after sowing) stage. For the purpose, a root auger measuring an 8-cm diameter and 15-cm length (core volume = 754.28 cm^3^) was utilized for root sampling up to the depth of 0–30 cm of the soil profile. The core ring of auger was maintained at the base level of the stem at the center. By gradually loosening the soil, the roots were collected. To prevent loss of finer roots while washing, the remaining soil adhered on roots was removed by gentle washing and placing the roots on sieve containers and immediately kept for refrigeration at 4°C. The root parameters, such as root length and the root surface area, were estimated from a computer-based RHIZO device scanner, which scans and analyzes the images using WIN-RHIZO software. These values were further used for calculating the root length density (RLD), root surface density (RSD), and root volume density (RVD) on the basis of unit soil volume in a cubic centimeter (cc). After the scanning, the root samples were subjected to drying at 60°C for 2 days for obtaining root dry weight (RDW). Number of nodules per plant was recorded by selecting five plants randomly at the flowering and maturity stage from the rows subjected for sampling from each plot. Nodules detached from the roots and the number of nodules were measured from each plant and expressed as the number of the nodules plant^–1^.

### Statistical analysis and data visualization

All the experimental data for various characters were statistically studied through the ANOVA method as described by Panse and Sukhatme (1985). The comparison of treatment means was made by the least significant difference (LSD) test at the probability level of *p* < 0.05 (Steel et al., 1997). The significance of treatment response was analyzed with the help of the ‘F’ (variance) test, and significance of difference was analyzed and critical difference (CD) or LSD calculated by using formula:


LSD⁢or⁢CD=2⁢MSE×t⁢at⁢ 5%⁢significance⁢leveln

where ‘MSE’ is the mean square error, ‘n’ is the number of observations, ‘t’ is the value of percentage point of ‘t’ distribution for error degrees of freedom at the 5% level of significance.

### Pearson correlation coefficient and multivariate analysis

Pearson correlation coefficient analysis was carried out to understand the association between diverse soil physical, chemical, and biological parameters. The R software (version 3.5.1) was used to perform Pearson correlation coefficient analysis. Two chemometric approaches *viz*. principal component analysis (PCA) and agglomerative hierarchical clustering (AHC) were used, resorting to SPSS of version 22. The cluster analysis (CA) was done, which supports in grouping the objects (cases) into classes (clusters) and depends on relative similarities within a class and dissimilarities between different classes. The findings of cluster analysis help in the interpretation of data and indicating the pattern and were presented as the heat map (Vega et al., 1998). A heat map represents two-dimensional illustration of data where values are analyzed by colors, which deliver the visual information. The heat map sorts the rows, and columns depend on the hierarchical clustering/cluster analysis. The colors will then be apportioned to the soil properties to represent the values. Principal component analysis (PCA) is a multivariate technique, which analyzes the data through various inter-correlated and quantified dependent variables to find out the abstract of the significant information from the data to represent it as a set of novel orthogonal variables referred to as principal components (PC), and to show the configuration of relationship among the observations and the variables (Mishra et al., 2017). For clarity in interpretation, data were resorted to Varimax orthogonal rotation.

## Results and discussion

### Plant growth and yield of pigeonpea

Effect of microbial-inoculants (MI)-mediated nutrient management on plant height of pigeonpea was not established significant at 30 DAS; however, it showed significance at 60 and 90 DAS and at the maturity stage (Table 4). Significantly higher plants height of pigeonpea at 60 and 90 DAS and the harvest stage were recorded with VC + MI treatment, which remained at par with the FYM + MI and LC + MI. Plant height at 60 and 90 DAS, and the harvest stage did not vary significantly with the application of RDF + MI over sole RDF. Among the organic sources, VC recorded higher plant height followed by FYM and LC under both treated and sole application. The DMA was significantly affected because of various treatments during 60 and 90 DAS and at the harvest stage, whereas, at 30 DAS, it was non-significant (Table 4). The effect of inoculation with organic resources was substantial over sole organic resources. However, the application of RDF + MI and sole RDF did not differ significantly with respect to DMA at 60 and 90 DAS and at the harvest stage. DMA with VC + MI application at 60 and 90 DAS and the harvest stage was at par with the use of FYM + MI and LC + MI. The DMA was higher in VC over FYM and LC. Significantly higher DMA was recorded with VC + MI over FYM + MI and LC + MI. Branches per plant of pigeonpea were influenced significantly due to the bio-inoculation of the crop with microbial inoculants (Table 4). The higher number of branches per plant (23.3) was noted with the use of VC + MI, which was at par with branching with FYM + MI. Inoculation of pigeonpea with MI was observed more effective when applied along with organic nutrient sources; however, integration of MI with RDF was found ineffective to enhance the branching significantly. The advantage of integrating MI with nutrient sources was to the tune of 47.9, 41.3, 25.9, and 2.5% for LC + MI, FYM + MI, VC + MI, and RDF + MI, respectively. LAI of pigeonpea was significantly affected due to MI-mediated nutrient management during 60 and 90 DAS, whereas, at 30 DAS, the effect was non-significant during both years (Table 4). Significantly, higher LAI at 60 and 90 DAS was found in RDF + MI; however, it was at par with the sole use of RDF. Higher LAI was measured with VC + MI application at 60 and 90 DAS and at the harvest stage, which was at par with the LAI under FYM + MI and LC + MI treatment during both years. Among the organic sources of nutrients, VC has resulted in the highest LAI, followed by FYM and LC under both treated and sole application.

**TABLE 4:** Influence of microbial inoculants-mediated nutrient management on crop growth parameters (mean of 2-year data) of pigeonpea.

Treatments*	Plant height (cm)				DMA (g plant^–1^)				Branches plant^–1^	LAI			CGR (g plant^–1^ day^–1^)		
					
	30 DAS	60 DAS	90 DAS	Maturity	30 DAS	60 DAS	90 DAS	Maturity	At 50% flowering (75 DAS)	30 DAS	60 DAS	90 DAS	0-30 DAS	30-60 DAS	60-90 DAS
Control	36.3	87.3^d^	107.2^d^	114.3^d^	4.0	10.2^e^	35.5^e^	46.8^e^	12.0^f^	0.32	0.94^d^	2.02^d^	1.11	1.76^e^	7.0^c^
RDF	38.3	98.5^c^	119.6^c^	131.1^c^	4.6	12.1^d^	40.5^d^	54.0^d^	16.2^d^	0.33	1.10^c^	2.26^c^	1.28	2.10^de^	8.0^bc^
RDF + MI	38.9	100.3^c^	120.9^c^	133.7^c^	4.7	12.4^d^	40.8^d^	55.2^d^	16.6^d^	0.35	1.12^c^	2.28^c^	1.30	2.19^d^	8.0^bc^
VC	38.2	107.8^b^	131.2^b^	145.9^b^	4.3	14.0^c^	44.4^c^	59.8^c^	18.5^c^	0.35	1.23^b^	2.50^b^	1.20	2.82^c^	8.7^ab^
FYM	37.2	97.7^c^	118.5^c^	130.6^c^	4.2	12.2^d^	40.0^d^	53.3^d^	15.5^de^	0.32	1.05^c^	2.24^c^	1.16	2.25^d^	7.8^bc^
LC	37.0	95.6^c^	115.8^c^	127.7^c^	4.2	11.8^d^	39.3^d^	52.3^d^	14.0^e^	0.35	1.05^c^	2.23^c^	1.15	2.18^d^	7.6^c^
VC + MI	39.0	119.4^a^	146.3^a^	165.1^a^	4.8	17.3^a^	50.5^a^	70.8^a^	23.3^a^	0.37	1.37^a^	2.77^a^	1.32	3.69^a^	9.5^a^
FYM + MI	39.3	117.0^a^	143.8^a^	162.9^a^	4.7	15.8^b^	47.7^b^	65.2^b^	21.9^ab^	0.35	1.36^a^	2.74^a^	1.30	3.23^ab^	9.2^a^
LC + MI	38.5	115.7^a^	141.5^a^	157.8^a^	4.8	15.5^b^	47.4^b^	64.7^b^	20.7^b^	0.34	1.33^a^	2.72^a^	1.33	3.16^bc^	9.1^a^
SEm±	1.1	1.8	2.6	3.5	0.3	0.3	0.8	1.3	0.6	0.01	0.03	0.06	0.07	0.13	0.4
LSD (*P* = 0.05)	NS	5.3	7.7	10.4	NS	1.0	2.5	4.0	1.7	NS	0.09	0.19	NS	0.40	1.1

Values in the same column followed by different letters are significantly different at *p* < 0.05 according to Duncan’s multiple-range test for separation of means.

*Refer to Table 2 for treatment description.

Effect of various MI-mediated nutrient management practices on crop growth rate ($\bar{C\,G\,R}$) was significant during 30–60 and 60–90 DAS (Table 4). Maximum $\bar{C\,G\,R}$ was recorded with VC + MI application at 30–60 and 60–90 DAS, which was at par with the FYM + MI and LC + MI. Significantly higher $\bar{C\,G\,R}$ was measured in inoculated organic sources of nutrients. Growth attributes *viz*. plant height, DMA, Branching, LAI, and $\bar{C\,G\,R}$, were established significantly better under MI-treated organic resources over sole organic resources of nutrients, which might be because of their PGP ability, meager competition for growth factors, high root growth, better soil aeration and microbial activity, and available nutrients in the long run might be responsible for superior growth attributes of the plant (Dhandayuthapani et al., 2015). The maximum value of growth attributes under VC + MI over other manures supports the fact of its nutrient richness (Table 3). In comparison to chemical fertilizers, vermicompost promotes biological fertility of soil through accessing resources (dissolved organic carbon and dissolved organic nitrogen) and environment for better microbial growth (Zhang et al., 2012). Hence, the higher contents of available nutrients and favorable microbial properties under VC + MI might have resulted in higher growth attributes over other manures (Song et al., 2015). The effectiveness of integrating microbial agents and vermicompost to replace for regular chemical fertilization practices is proved (Song et al., 2015). Improvement in growth attributes under inoculated VC treatment over sole VC and other inoculated manures was due to the better nutrient management through the integrated use of microbial-inoculants, which raised the availability of major nutrients to plants, which further result in elevation of biochemical and physiological processes *viz*., energy transfer reactions, root development, and photosynthesis, which allowed the crop to grow to their full potential (Shete et al., 2010; Kumar et al., 2015; Zeyad et al., 2021; Sharma et al., 2022).

### Root growth and development

The MI-mediated nutrient management significantly affected the root length (RL) and RLD (Table 5). The influence of inoculation with RDF was non-significant over sole RDF, while organic sources, along with MI, had significant effect over sole use of organic sources of nutrients. FYM caused higher RL followed by LC and VC under both MI-treated and sole applications. FYM + MI showed the highest RL and RLD, which was at par with LC + MI, as well as sole FYM. Inoculation response was seen higher with LC + MI (18%), followed by 16.7, 8.2, and 5.8%, with VC + MI, FYM + MI, and RDF + MI, respectively. RDW was noted to be influenced significantly when the organic as well as inorganic nutrient sources were integrated with MI cultures (Table 5). However, the response of integration was higher (29%) with VC + MI, followed by LC + MI (28%), RDF + MI (12.9%), and FYM + MI (9.5%), although the FYM + MI recorded the highest (41.5 g) RDW, and this was at par by the use of LC + MI and FYM + MI. RSD was recorded to be influenced significantly when the organic, as well as inorganic nutrient sources, were integrated with MI cultures (Table 5). However, the response of integration was higher (29.4%) with VC + MI, followed by LC + MI (28.5%), FYM + MI (16.6%) and RDF + MI (10.5%), although the FYM + MI observed the highest RSD (40.8 cm^2^cc^–1^), and it was at par with LC + MI and FYM + MI. The RSD was ranked as FYM + MI ≈ LC + MI > VC + MI ≈ LC ≈ FYM > VC > RDF + MI ≈ RDF > control. RVD was enhanced with the integration of MI with the organic and inorganic nutrient sources; however, this effect was not significant (Table 5). Among the organic combinations, FYM + MI recorded the highest (16.1 cm^3^ cc^–1^) and LC + MI the lowest value (14.1 cm^3^ cc^–1^) of RVD and VC + MI as intermediate (15.2 cm^3^ cc^–1^). Number of the nodules plant^–1^ was measured at the maximum flowering and maturity stage of the pigeonpea crop, and it was noticed that MI-mediated nutrient management practices significantly influenced the nodulation (Table 5). Organic sources of nutrients, especially FYM, resulted in a higher number of root nodules when applied as sole or in combination with MI. However, LC (sole), as well as LC + MI performed at par with FYM + MI, while VC (sole), as well as VC + MI could not perform statistically at par with FYM and LC. Integration effect of MI with various nutrient sources on nodulation was 46.1, 31.5, 24, and 23% higher with LC, FYM, RDF, and VC, respectively, at the flowering stage, while, at the maturity, it followed the trend as RDF + MI (82.1%) > FYM + MI (57.9%) > VC + MI (56.3) and LC + MI (55.4%).

**TABLE 5:** Influence of microbial inoculants-mediated nutrient management on rhizospheric attributes (mean of 2-year data) of pigeonpea.

Treatments*	Root length (cm)	Root length density (cm cc^–1^)	Root dry weight (g)	Root surface density (cm^2^ cc^–1^)	Root volume density (cm^3^ cc^–1^)	Root nodules (no.)	
						
						Flowering	Maturity
Control	34.1^f^	34.2^d^	22.7^f^	23.1_*f*_	9.4^f^	12.6^f^	6.5^f^
RDF	37.8^e^	37.9^c^	28.7^e^	26.8^e^	11.3^ef^	19.2^cd^	8.4^e^
RDF + MI	40.0^d^	39.7^c^	32.4^d^	29.6^de^	11.9^def^	23.8^b^	15.3^b^
VC	38.4^de^	38.6^c^	30.4^de^	28.9^de^	13.7^bcd^	16.5^e^	8.7^e^
FYM	43.9^c^	44.0^b^	37.9^c^	34.9^c^	15.1^abc^	20.0^cd^	11.4^d^
LC	38.9^de^	39.0^c^	31.4^de^	30.2^d^	12.9b^cde^	17.8^de^	11.2^d^
VC + MI	44.8^bc^	45.3^ab^	39.2^bc^	37.4^bc^	15.2^ab^	20.3^c^	13.6^c^
FYM + MI	47.5^a^	48.0^a^	41.5^a^	40.7^a^	16.1^a^	26.3^a^	18^a^
LC + MI	45.9^b^	46.6^ab^	40.2^b^	38.8^ab^	14.0^cde^	26.0^ab^	17.4^a^
SEm±	1.1	1.0	1.5	1.0	0.6	0.76	0.57
LSD (*P* = 0.05)	3.2	3.0	4.4	3.1	1.8	2.28	1.72

Values in the same column followed by different letters are significantly different at *p* < 0.05 according to Duncan’s multiple-range test for separation of means.

*Refer to Table 2 for treatment description.

The resultant favorable rhizospheric attributes with the application of FYM followed by VC > LC > RDF under both inoculated and un-inoculated treatments might be due to the better soil properties, especially, SOC, WSA, water holding capacity, lower BD, higher porosity, and higher microbial activity, which created easiness for the proper development of roots (Dar et al., 2017; Singh et al., 2020a; Kumar et al., 2022). Another reason for higher root development under FYM + MI might be the accelerated production of PGP and root growth-promoting hormones by microbial inoculants, such as IAA, phosphatase, and ACC deaminase (Helman et al., 2012). According to a report, there was a significant improvement in root volume (62%), root weight (74%), root length (54%), and root area (75%) due to MI inoculation (Mäder et al., 2011; Kumar et al., 2015). Improvement in rooting characteristics under bulky organic manures may also be due to the synergistic effect of certain microbes that enable organic manures to mineralize quickly and release plant nutrients in a balanced and quick manner (Gupta et al., 2020). FYM, being rich in organic carbon, provides favorable growth conditions for plant growth-promoting rhizobacteria (PGPR), which shows a symbiotic relationship with plant roots and enables them to uptake more nutrients and water that ultimately helps in the optimum development of the root rhizosphere (Paramesh et al., 2020; Kumar A. et al., 2021). FYM, along with microbial inoculants, increases organic carbon, available nutrients, soil biological properties *viz*., dehydrogenase activity and improved microbial population (Kumar A. et al., 2021; Kumar S. et al., 2021), which becomes the reason for the enhanced root nodulation and root growth attributes (Singh et al., 2020b,^2021^). Inoculated organic treatments increase the porosity of soil, which results in an improvement in root growth and raises soil biological/microbial activities, which enhance oxygen/air availability in the region of plant roots (Dar et al., 2017).

### Soil physical properties

**FIGURE 2:**
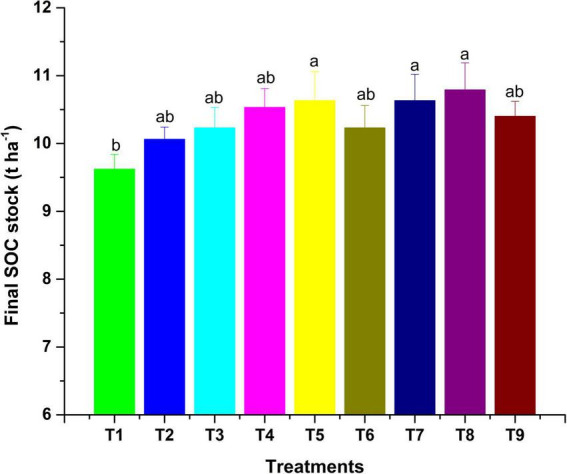
Final SOC stock as influenced by MI-mediated nutrient management. Different letters in alphabetical orders in figure are generally assigned to indicate the significantly different values at *p* < 0.05 according to Duncan’s multiple-range test for separation of means.

The effect of MI-based nutrient management along with organic sources or RDF on BD of soil was non-significant (*p* < 0.05) (Table 6). However, a slight reduction in BD was observed in plots applied with organic manures. A maximum reduction in the value of BD was found over the initial value of 1.58 g cc^–1^ in the FYM + MI treatment, while the least influence was recorded in the control plot. The efficacy of organic nutrient management practices in improving BD can be ranked as FYM > LC > VC. Soil aggregation expressed in terms of percentage of geometric mean diameter (GMD), mean weight diameter (MWD), and water-stable aggregates (WSA) is presented in Table 6. Significantly (*p* < 0.05), the higher WSA (22.0%), MWD (1.13 mm), and GMD (0.93 mm) were recorded in treatment FYM + MI. The effect of inoculation with microbial inoculants was significant (*p* < 0.05) in the case of RDF, FYM, VC; however, inoculation with LC was non-significantly effective. The extent of this improvement in WSA under inoculated treatments over the sole application was 7.7, 4.4, and 3.5% for FYM, VC, and RDF, respectively; in MWD: 8.8, 19.8, and 11.2%; in GMD: 11.8, 14.4, and 17.6%, respectively. Improvement in aggregates stability under LC + MI application was found to be inefficient. Significant improvement in WSA, MWD, and GMD under MI-mediated organic combinations, especially FYM + MI, might be due to a favorable impact on soil aeration as a result of proper rhizosphere development (Kumar S. et al., 2021) and optimum microbial activity (Singh et al., 2017; Kumar et al., 2018b; Kumar A. et al., 2021). The enhanced level of organic matter under organic treatments also played an important role in raising soil physical health (Nandapure et al., 2011), as is evident from the correlation matrix (Table 7) and the heat map analysis (Figure 2), which indicates a strong relationship between soil properties. Another reason for the improvement of soil physical properties under FYM + MI could be due to maintained soil-plant-water relationship in a better way as a result of proper root development and soil aggregation (Kumar et al., 2015; Graham et al., 2017; Singh et al., 2020b; Bhakar et al., 2021; Kumar A. et al., 2021). Accordingly, soils managed with organic manure, along with MI, tend to have more stable soil aggregates in comparison to inorganic fertilizers and MI combinations (Babu et al., 2015).

**TABLE 6:** Influence of microbial inoculants-mediated nutrient management on soil physical, chemical, and biological properties (at the end of the 2-year experiment).

Treatments*	Soil physical properties				Soil chemical properties				Soil biological properties (During flowering stage, 70 DAS)				
			
	BD (Mg m^–3^)	WSA (%)	MWD (mm)	GMD (mm)	Available N (kg ha^–1^)	Available P (kg ha^–1^)	Available K (kg ha^–1^)	OC (%)	DHA activity (μg TPF g^–1^ soil day^–1^)	APA activity (μg p-nitro phenol g^–1^ soil hr^–1^)	SMBC (mg kg^–1^ soil)	ARA (n moles ethylene gram^–1^ soil hr^–1^)	Polysaccharides (mg kg^–1^ soil)
Control	1.59	10.7^g^	0.59^d^	0.48^e^	161.7^b^	10.7^c^	276.5^d^	0.40	110.3^e^	54.7^e^	117.3^e^	18.4^e^	315.0^f^
RDF	1.59	16.7^d^	0.74^cd^	0.56^e^	165.0^b^	16.7^b^	325.3^bc^	0.42	132.3^d^	72.1^d^	141.5^d^	21.6^de^	338.3^e^
RDF + MI	1.58	17.3^c^	0.84^bcd^	0.68^d^	167.3^b^	17.3^b^	335.5^abc^	0.43	136.7^d^	78.3^d^	146.1^cd^	22.9^cd^	368.3^cd^
VC	1.56	15.3^f^	0.89^abc^	0.77^cd^	187.3^a^	20.3^a^	334.7^abc^	0.45	156.4^c^	92.7^c^	160.6^c^	23.6^cd^	367.6^d^
FYM	1.54	20.3^b^	1.03^ab^	0.82^abc^	182.0^a^	16.7^b^	325.5^bc^	0.46	180.3^b^	111.7^b^	193.1^b^	25.1^bc^	383.0^c^
LC	1.55	16.7^d^	0.99^abc^	0.74^cd^	181.0^a^	15.3^b^	321.4^c^	0.45	176.4^b^	107.1^b^	188.5^b^	23.9^cd^	370.6^cd^
VC + MI	1.54	16.0^e^	1.11^a^	0.90^ab^	191.0^a^	22.0^a^	346.5^a^	0.46	183.3^b^	113.7^b^	197.3^b^	24.7^bcd^	400.6^b^
FYM + MI	1.53	22.0^a^	1.13^a^	0.93^a^	183.0^a^	17.0^b^	340.6^ab^	0.46	208.1^a^	132.3^a^	222.3^a^	29.5^a^	419.0^a^
LC + MI	1.54	17.0^cd^	1.00^ab^	0.80^bc^	186.0^a^	16.0^b^	335.0^abc^	0.45	203.7^a^	128.1^a^	217.0^a^	27.6^ab^	405.3^ab^
SEm±	0.03	0.21	0.09	0.04	4.2	1.0	6.0	0.03	6.1	4.6	5.9	1.1	3.9
LSD (*P* = 0.05)	NS	0.64	0.26	0.11	12.4	3.0	18.1	NS	18.2	13.7	17.6	3.4	15.8

Values in the same column followed by different letters are significantly different at *p* < 0.05 according to Duncan’s multiple-range test for separation of means.

*Refer to Table 2 for treatment description.

**TABLE 7:** Correlation coefficient (r)* matrix between soil physical, chemical, and biological properties.

	MWD	GMD	BD	WSA	OC	N	P	K	DH	APA	MBC	ARA	POLY
MWD	***1***	***0.933***	–***0.867***	**0.681**	***0.898***	***0.895***	**0.591**	***0.737***	***0.942***	***0.95***	***0.941***	***0.868***	***0.938***
GMD	***0.933***	***1***	–**0.672**	**0.682**	***0.813***	***0.883***	***0.753***	***0.839***	***0.865***	***0.879***	***0.854***	***0.863***	***0.943***
BD	–***0.867***	–**0.672**	***1***	–**0.568**	–***0.791***	–***0.702***	–0.192	–0.405	–***0.887***	–***0.883***	–***0.906***	–***0.742***	–***0.779***
WSA	**0.681**	**0.682**	–**0.568**	***1***	0.473	0.408	0.36	**0.68**	***0.715***	***0.726***	***0.722***	***0.828***	***0.752***
OC	***0.898***	***0.813***	–***0.791***	0.473	***1***	***0.951***	0.473	***0.522***	***0.869***	***0.871***	***0.849***	***0.738***	***0.771***
N	***0.895***	***0.883***	–***0.702***	0.408	***0.951***	***1***	**0.683**	**0.667**	***0.825***	***0.832***	***0.804***	***0.705***	***0.796***
P	**0.591**	***0.753***	–0.192	0.36	0.473	**0.683**	***1***	***0.864***	0.41	0.432	0.393	0.421	**0.56**
K	***0.737***	***0.839***	–0.405	**0.68**	***0.522***	**0.667**	***0.864***	***1***	**0.667**	**0.684**	**0.659**	***0.737***	***0.801***
DH	***0.942***	***0.865***	–***0.887***	***0.715***	***0.869***	***0.825***	0.41	**0.667**	***1***	***0.999***	***0.998***	***0.952***	***0.948***
APA	***0.95***	***0.879***	–***0.883***	***0.726***	***0.871***	***0.832***	0.432	**0.684**	***0.999***	***1***	***0.997***	***0.955***	***0.957***
MBC	***0.941***	***0.854***	–***0.906***	***0.722***	***0.849***	***0.804***	0.393	**0.659**	***0.998***	***0.997***	***1***	***0.947***	***0.947***
ARA	***0.868***	***0.863***	–***0.742***	***0.828***	***0.738***	***0.705***	0.421	***0.737***	***0.952***	***0.955***	***0.947***	***1***	***0.958***
POLY	***0.938***	***0.943***	–***0.779***	***0.752***	***0.771***	***0.796***	**0.56**	***0.801***	***0.948***	***0.957***	***0.947***	***0.958***	***1***

The correlation coefficient (r) values are significantly positive at p < 0.01 (Boldfaced italics) and p < 0.05 (Bold) levels of probability (two-tailed).

Boldfaced yellow italics and bold blue-colored fonts indicate significantly negative correlation at p < 0.01 and p < 0.05 levels of probability (two-tailed).

*Correlation coefficient (r) values correspond directly to the color code from (decrease) green to yellow and red, respectively.

MWD, mean weight diameter; GMD, geometric mean diameter; BD, bulk density; WSA, water stable aggregates; OC, organic carbon; N, nitrogen; P, phosphorus; K, potassium; DH, dehydrogenase; APA, alkaline phosphatase activity; MBC, microbial biomass carbon; ARA, acetylene reduction activity, POLY, total polysaccharides.

### Soil chemical properties

**FIGURE 3:**
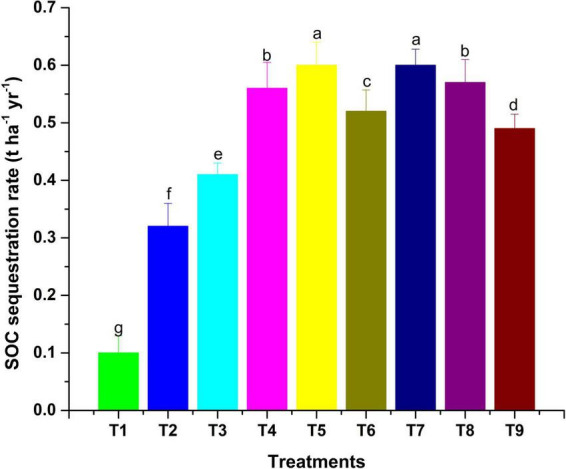
The SOC seq. rate as influenced by MI-mediated nutrient management. Different letters in alphabetical orders in figure are generally assigned to indicate the significantly different values at *p* < 0.05 according to Duncan’s multiple-range test for separation of means.

The observations on available-N status in soil revealed that integration of microbial inoculants with organic and inorganic sources of plant nutrients was ineffective for showing a significant response on the status of available nitrogen in soil (Table 6). However, sole and integrated organic sources were significantly effective for impacting available nitrogen in soil positively. The extent of additional nitrogen added by the application of organic sources, along with MI, as compared to RDF + MI was 12.4% higher with VC + MI, 8.1% with FYM + MI and 10.1% with LC + MI. Hence, this indicates that integration of MI with organic sources is significantly advantageous as compared to integration with chemical fertilizers. In comparison to sole application, the integration influence of RDF + MI, VC + MI, and LC + MI was higher to the tune of 1.4, 1.9, and 2.7%, respectively; however, FYM + MI was lesser effective by 0.5%. Available P status in soil was significantly (*p* < 0.05) affected by organic nutrient sources over inorganic sources (Table 6). The addition of available P in soil was analyzed to be higher with the use of VC + MI, and this was significantly higher than FYM + MI, LC + MI, RDF + MI, RDF, FYM, and LC; however, it was non-significant with sole VC application. The quantity of available P added in soil under VC + MI was 21.4% higher than RDF + MI; however, FYM + MI and LC + MI have added a lesser amount of available N in soil by -1.8% and -8.1%, respectively. In comparison to sole application, integration effect of RDF + MI, VC + MI, FYM + MI, and LC + MI was higher to the tune of 3.5, 7.7, 1.8, and 4.4%, respectively, with respect to available soil P. Status of available K in the soil at the end of a 2-year experiment was established significantly (*p* < 0.05) higher (Table 6) over the initial value (Table 1) under all the nutrient management treatments except control. Among the various MI-based combinations of nutrient management, application of VC + MI resulted in a significantly higher level of available K (346 kg ha^–1^) in comparison to FYM, LC, and RDF; however, it was at par with available K status under VC, FYM + MI, RDF + MI, and LC + MI treatment. The response of integration of MI with different nutrient sources over the sole application was higher with FYM + MI (4.4%), followed by LC + MI (4.2%), VC + MI (3.5%), and RDF + MI (3%). The non-significant deviation was observed due to MI-mediated nutrient management on SOC (Table 6). However, a relatively higher amount of SOC was added to the soil with the use of organic resources of nutrients over inorganic sources. Among the organic sources, FYM added a relatively higher amount of SOC in the soil when applied sole as well as in combination with MI. The SOC stock (Mg ha^–1^) was also observed higher under inoculated manure treatments (Figure 2). The trend of estimated final SOC stock was observed in the order of FYM + MI > FYM = VC + MI > VC > LC + MI > LC > RDF + MI > RDF > control. Observations in Figure 2 revealed that inoculation of crops with MI influenced the SOC stock; however, marginal improvement was noticed when manures or fertilizers were applied in combination. The SOC sequestration rate (t ha^–1^ yr^–1^) also showed almost similar findings (Figure 3).

The improvement in the level of available NPK under VC + MI application might be due to enhanced nutrient release as a result of microbes-mediated accelerated decomposition and mineralization of inoculated manure (Kuotsu et al., 2014; Gupta et al., 2020). Application of inoculated manure proved to encourage and sustain soil health by raising the SOC, available NPK status, and uptake of nutrients by plants by reducing the BD of soil (Tiwari et al., 2011; Kumar et al., 2015, 2018a; Kumar S. et al., 2021). SOC, SOC Stock, and the SOC sequestration rate were found to be influenced in a non-significant way, following the mechanism of soil buffering capacity, which shows that a soil resists the changes in its properties up to a certain period, owing to the buffering ability (Yigini and Panagos, 2016). Hence, this 2-year experiment was not sufficient enough to cause a significant effect on SOC; however, a slight improvement was definitely noted under organic combinations, which might be due to the higher addition of SOM and optimum mineralization of this SOM under the microbial-rich environment in root rhizosphere (Snapp et al., 1998; Tiwari et al., 2011; Thapa et al., 2017).

### Soil biological properties

Bio-inoculation of pigeonpea seeds with MI culture of N, P, and K, along with the treatment of organic manures (FYM, VC, LC), improved the dehydrogenase enzyme activity (μg TPF g^–1^ soil day^–1^) in soil, significantly; however, RDF + MI was non-significantly effective to influence dehydrogenase activity over the sole RDF (Table 6). The trend of dehydrogenase activity under various treatments was FYM + MI ≈ LC + MI > VC + MI ≈ LC ≈ FYM > VC > RDF + MI ≈ RDF > control. However, the increment of this positive influence due to bio-inoculation of nutrient sources over the sole application was to the tune of 17.2, 15.5, 15.4, and 3.3% under VC + MI, LC + MI, FYM + MI, and RDF + MI, respectively. Hence, it can be reported that MI with organic manures may affect the activity of dehydrogenase enzymes in soil significantly. Alkaline phosphatase activity of soil was also influenced significantly when the pigeonpea crop was inoculated with microbial strains of N, P, and K fixers and solubilizers (Table 6). Among the various MI-based nutrient management practices, use of FYM + MI resulted in higher activity (132.3 μg p-nitro phenol g^–1^ soil hr^–1^) of alkaline phosphatase enzyme in soil; however, LC + MI also exhibited almost similar value (128.1 μg p-nitro phenol g^–1^ soil hr^–1^). The trend of influence on alkaline phosphatase activity under the experimentation was seen in the order of FYM + MI ≈ LC + MI > VC + MI ≈ LC ≈ FYM > VC > RDF + MI ≈ RDF > control. However, the augmentation of this positive impact under integrated treatment over the sole application was to the tune of 22.7, 19.6, 18.4, and 8.6% under VC + MI, LC + MI, FYM + MI, and RDF + MI, respectively. SMBC content in soil was affected significantly (*p* < 0.05) due to MI-mediated nutrient management (Table 6). Among various treatments, application of FYM + MI resulted in higher SMBC (222.3 mg kg^–1^ soil), which was at par with SMBC content of 217.-mg kg^–1^ soil under LC + MI application. The ranking of impact on SMBC under the experimentation was in the order of FYM + MI ≈ LC + MI > VC + MI ≈ LC ≈ FYM > VC > RDF + MI ≈ RDF > control. However, the incremental effect of inoculation was noted as 22.9, 15.1, 15.1, and 3.3% under VC + MI, LC + MI, FYM + MI, and RDF + MI, respectively, over the sole application of these nutrient sources, which profound that inoculation of microbial strains with organics may be more advantageous over the inorganic when SMBC is concerned. Acetylene reduction activity of soil was estimated in the range of 18.4–29.5 n moles ethylene gram^–1^ soil hr^–1^. The higher value of ARA (29.5 n moles ethylene gram^–1^ soil hr^–1^) was measured by the combined use of FYM + MI, which was found higher significantly (*p* < 0.05) than ARA under the remaining treatments. The order of influence was noted as FYM + MI > LC + MI > FYM > VC + MI > VC ≈ RDF + MI ≈ RDF > control. The advantage of integrating MI with nutrient sources was to the tune of 17.5, 15.5, 6.0, and 4.7% for FYM + MI, LC + MI, RDF + MI, and VC + MI, respectively. This inferred that ARA activity response was highest with FYM + MI and lowest with VC + MI application. MI-mediated nutrient management practices affected the total polysaccharides content in soil significantly (Table 6). The highest value (419 mg kg^–1^ soil) of total polysaccharides was measured by the use of FYM + MI, which was significantly (*p* < 0.05) higher than others. The sequence of influence due to the imposition of different treatments was noted in the order of FYM + MI > LC + MI > VC + MI > LC ≈ RDF + MI ≈ VC ≈ RDF + MI > RDF > control. The improvement of integrating MI with nutrient sources was to the tune of 9.4, 9.4, 9. and 8.9% for FYM + MI, LC + MI, RDF + MI, and VC + MI, respectively.

The resulted trend of soil microbial parameters *viz.* dehydrogenase, alkaline phosphatase, SMBC, acetylene reduction activity, and the total polysaccharides level followed the order as FYM > LC > VC with and without MI, and these might be due to the improvement in soil-water relationship, enhanced addition of nutrients and organic carbon through microbes-mediated-accelerated decomposition of FYM (Samal et al., 2017; Ghosh et al., 2020). Inoculated manures enhance SOC and available moisture in the soil, which improves microbial health of the soil as microbial health is positively correlated with enhanced soil moisture status and organic carbon content in soil (Almeida et al., 2011). Organic manures promote the soil MBC and enzyme activities as compared to mineral fertilizers (Babu et al., 2020b). The soil phosphatase activity is recorded to enhance in rhizosphere by 2–3 times under inoculated treatments as that of un-inoculated soil (Gong et al., 2009). Enhancement in soil biological properties by adding the inoculated organic manures was due to higher population and activity of soil microbes and accelerated mineralization of organic manures, coupled with better soil physical parameters, such as BD and soil aggregation (Bongiorno et al., 2019). The inhibitory effect on enzyme activity was found with higher levels of RDF, which may be due to interference of nitrate, which serves as a substitute electron acceptor, ensuing in the drop down of enzyme activity (Babu et al., 2020a). The application of organic sources of nutrients increased organic carbon status in soil, and it acts as a substrate for microorganisms and also increased the availability of nutrients required for growth of microorganisms, resulting in higher microbial activity and, in turn, corresponded with higher SMBC and population counts in soil (Mäder et al., 2011). Increased soil enzymatic activities under inoculated organic manures application might be due to improvement in microbial population, which increased soil enzyme activities and thereby soil health (Kumar S. et al., 2021).

### Correlation coefficient (*r*) matrix between soil physical, chemical, and biological characteristics

The univariate Pearson’s correlation coefficient (*r*) matrix (Table 7) indicated that physico-chemical and biological properties of soil were found to be significant and positively correlated among them. The MWD was substantially and positively correlated among physico-chemical and biological characteristics of soil; however, it had a highly substantial negatively correlated (*p* < 0.01 and *r* = –0.867) with BD. The GMD exhibited a substantial negatively correlated (*p* < 0.01 and *r* = –0.672) with BD; however, it showed a positive correlation with the rest of the parameters. BD displayed a highly significant negative correlation (*p* < 0.01) among physic-chemical and biological properties, while the correlation with WSA displayed a substantial negative correlation (*p* < 0.05). WSA noticed a significant correlation with the following descending order ARA > POLY > APA > MBC > OC > DH > K. Organic carbon recorded a highly significant positive correlation (*p* < 0.01) with these parameters in descending order N > APA > Poly > DH > MBC > ARA > K > P. N noticed a highly significant negative correlation (*p* < 0.01) with K, DH, APC, MBC, ARA, and POLY and a significant positive correlation (*p* < 0.05) with P. P also recorded a significant positive correlation with K (*p* < 0.01 and *r* = 0.864) and POLY (*p* < 0.05 and *r* = 0.560), while K was significantly correlated (*p* < 0.01) with DH, APA, MBA, ARA, and POLY (*p* < 0.05) biological properties. DH exhibited a substantial positive correlation (*p* < 0.01) with APA, MBC, ARA, and POLY. Similarly, APA displayed a substantial positive correlation (*p* < 0.01) with MBC, ARA, and POLY. MBC was positively and substantially correlated (*p* < 0.01) with ARA and POLY. ARA also displayed a substantial positive correlation (*p* < 0.01 and *r* = 0.958) with POLY.

### Principal component analysis of physical, chemical, and biological properties

**FIGURE 4:**
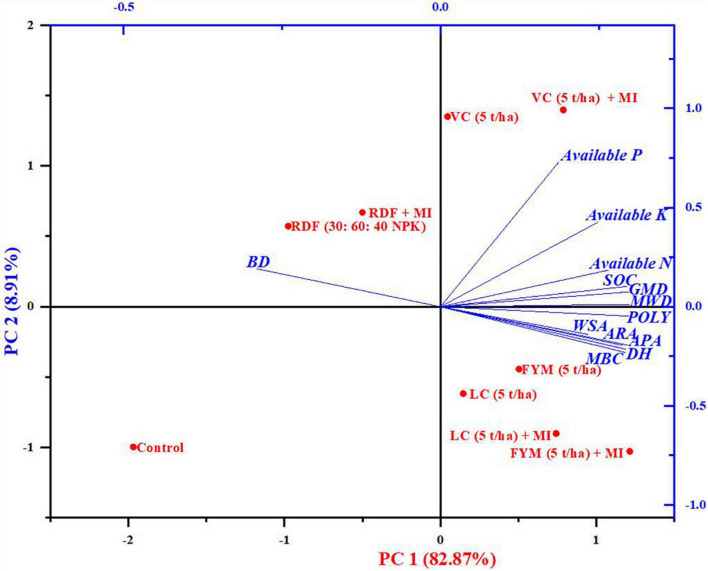
The two-dimensional graphical biplot showing both the loading and the scoreplot of soil physical, chemical, and biological properties.

The results based on principal component analysis conducted on the physical, chemical, and biological characteristics of soil extracted two dominant principal components: PC1 and PC2, accounting for 79.20 and 10.30% variability, which explained up to 90% of the total variability (Figure 4). The biplot (Figure 4) depicted both factors loading of physical, chemical, and biological characteristics of soil (Blue color) and the score of treatment (Red color). The loading plot of the biplot (Figure 4) displayed that PC1 had large positive loadings on MBC, which was closely followed by DH, APA, ARA, and POLY, which belong to soil biological properties; however, it shows negative loading on BD. Furthermore, these parameters are strictly correlated to each other as the angle amid the variables of 0 or 1,800 divulges a correlation of 1 or -1, respectively (Kohler and Luniak, 2005). Similarly, PC 2 also exerted strong positive loading on P, K, and N, and negative loading on BD again. In respect of the scoreplot of the biplot (Figure 4), it can be seen that treatments, including VC + MI, occupied the Ist quadrant of the score plot and significantly influenced the component of PC2 (P, K, N, SOC, MWD), which were dominantly soil chemical and physical characteristics. Similarly, in the second quadrant, the treatments RDF + MI and RDF affected the BD of soil; however, the control plot (third quadrant) did not have any substantial impact on any parameters of soil. Interestingly, in the fourth quadrant, the treatments, including FYM (5 t ha^–1^), LC, LC + MI, and FYM + MI, substantially affected most components of PC1 *viz*., soil biological properties, comprising of MBC, DH, ARA, and POLY (Figure 4).

### Heat map depicting the clustering of chemical, physical, and biological properties

**FIGURE 5:**
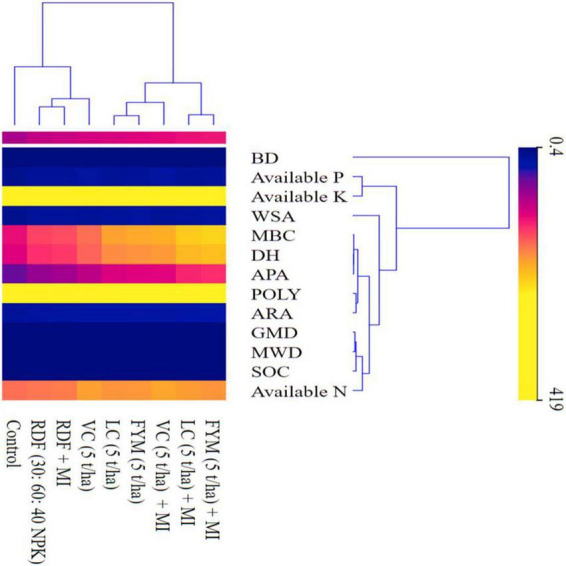
The heat map depicting the clustering of soil physical, chemical, and biological properties. MWD, mean weight diameter; GMD, geometric mean diameter; BD, bulk density; WSA, water stable aggregates; OC, organic carbon; N, nitrogen; P, phosphorus; K, potassium; DH, dehydrogenase; APA, alkaline phosphatase activity; MBC, microbial biomass carbon; ARA, acetylene reduction activity; POLY, total polysaccharides.

The heatmap generated (Figure 5) displayed the soil properties vertically and treatments horizontally wherein three dominant clusters were formed. The 1st cluster consists of primary nutrients P and K, while the 2nd cluster consists of soil biological properties (MBC, DHA, APA, POLY, ARA). Similarly, the 3rd cluster encompasses root characters (RSD, RLD, RL, RDW, and RVD) and, finally, a pair of soil physical properties (GMD and MWD) and soil chemical properties (OC and N). BD was the only soil physical parameter that formed a discrete outlier without clustering with any other parameters under investigation, as it had a negative correlation with them.

## Conclusion

The need of integrated and balanced nutrient management was a rational for the experiment “microbes-mediated integrated nutrient management for improved rhizo-modulation, pigeonpea productivity, and soil bio-fertility in a semiarid agro-ecology.” The holistic findings concluded that the integration of certain N fixing, P, and K, solubilizing microbial inoculants along with organic manures could have significant impact on the crop growth, root development, and soil health parameters. A significant response of integration of MI with organic manures was assessed, following the trend VC + MI > FYM + MI > LC + MI > RDF + MI for plant shoot-root growth attributes and soil chemical health and bio-fertility. FYM + MI were exceptionally superior with respect to the soil physical health. Such findings were much better or at par with the results RDF + MI, which establish a base for the replacement of the chemical fertilizers in pigeonpea cultivation under pigeonpea-wheat crop rotation. Besides a solution for overuse of chemical fertilizers, study outcomes are also believed to solve the problem of crop residues and animal bi-products management by promoting the microbial consortia and organic manures-based agro-industries, which are basic to the microbial inoculants and organic manure-based INM. This way, outcomes of the study would add to the goal of sustainable agricultural development by producing quality crop produce, maintaining agro-biodiversity and making the soils healthy, fertile, and productive.

## Data availability statement

The raw data supporting the conclusions of this article will be made available by the corresponding author.

## Author contributions

GG, SD, MR, AcD, VS, and LS: conceptualization. GG, AdK, VS, DJ, LS, KK, SR, and PU: methodology. GG, IB, DJ, SD, MR, AmK, KK, and MS: formal analysis. GG, AdK, AuD, DJ, AC, and SD: investigation. SD, LS, VS, and AcD: resources. GG, SD, IB, DJ, AmK, and MS: data curation. GG, AmK, IB, DJ, AC, SR, and PU: writing–original draft. SD, AcD, SR, PU, PD, MR, VT, and EJ: suggestions, editing, and reviewing the manuscript. All authors read and agreed to the published version of the manuscript.
